# Rapidly fatal SMARCA4-deficient undifferentiated sarcoma originating from hybrid hemosiderotic fibrolipomatous tumor/pleomorphic hyalinizing angiectatic tumor of the foot

**DOI:** 10.1007/s00428-021-03167-6

**Published:** 2021-08-14

**Authors:** Abbas Agaimy, Norbert Meidenbauer, William R. Sukov, Robert Stoehr, Michael Vieth, Frank Roemer, Robert Grützmann, Andrew L. Folpe

**Affiliations:** 1 Institute of Pathology, Friedrich-Alexander University Erlangen-Nürnberg (FAU), Erlangen, Germany; 2 Departments of Internal Medicine 5 (Medical Oncology and Hematology), Friedrich-Alexander University Erlangen-Nürnberg (FAU), Erlangen, Germany; 3 Department of Laboratory Medicine and Pathology, Mayo Clinic, Rochester, MN USA; 4 Institute of Pathology, Klinikum Bayreuth GmbH, Bayreuth, Germany; 5 Institute of Radiology, Friedrich-Alexander University Erlangen-Nürnberg (FAU), Erlangen, Germany; 6 Department of Surgery, Friedrich-Alexander University Erlangen-Nürnberg (FAU), Erlangen, Germany

**Keywords:** SWI/SNF complex, Pleomorphic hyalinizing angiectatic tumor, PHAT, Hemosiderotic fibrolipomatous tumor, HFLT, SMARCA4, Undifferentiated sarcoma

## Abstract

Pleomorphic hyalinizing angiectatic tumor (PHAT) of soft parts and hemosiderotic fibrolipomatous tumor (HFLT) are two rare low-grade locally recurring neoplasms with predilection for the foot/ankle. Recent studies support a close link between the two entities, and origin of PHAT from HFLT and occurrence of hybrid HFLT/PHAT have been documented. Both lesions often harbor *TGFBR3* or *MGEA5* rearrangements. Rare sarcomas originating from HFLT/PHAT have been reported, typically resembling myxofibrosarcoma or myxoinflammatory fibroblastic sarcoma. We describe a novel SMARCA4-deficient undifferentiated sarcoma with rhabdoid features originating from hybrid HFLT/PHAT in the foot of a 54-year-old male. The tumor pursued a highly aggressive course with rapid regrowth after resection and multiple metastases resulting in patient’s death within 5 months, despite systemic chemotherapy. Immunohistochemistry revealed SMARCA4 loss in the undifferentiated sarcoma, but not in the HFLT/PHAT. Molecular testing confirmed *TGFBR3/MGEA5* rearrangements. This report expands the phenotypes of sarcomas developing from pre-existing PHAT/HFLT.

## Introduction

Pleomorphic hyalinizing angiectatic tumor (PHAT) is a rare locally recurring, non-metastasizing low-grade neoplasm with predilection for the foot/ankle, first described by Smith, Fisher and Weiss in 1996 [1]. PHAT is characterized by ectatic vessels filled by fibrin and surrounded by a cellular, CD34-positive proliferation of markedly pleomorphic but mitotically inactive cells containing abundant intracytoplasmic hemosiderin [1, 2]. Most cases present as subcutaneous swellings in the foot/ ankle region and only rarely as deep soft tissue masses at other sites [1, 2]. Hemosiderotic fibrolipomatous tumor (HFLT) is another rare low-grade neoplasm first described by Marshal-Taylor and Farnburg-Smith in 2000 as “hemosiderotic fibrohistiocytic lipomatous lesion,” and initially felt to be reactive in nature [3]. In 2006, Browne and Fletcher further delineated its clinicopathological features, convincingly established its neoplastic nature and coined the current name, “HFLT” [4]. Like PHAT, HFLT develops mainly in the ankle/foot region of middle-aged patients, and has a local recurrence rate of up to 50% [3, 4]. Histology shows bland, hemosiderin-containing spindle cells, some with nuclear pseudoinclusions, entrapping an increased amount of normal-looking fat and growing around small, damaged blood vessels [3, 4]. Both PHAT and HFLT may show stromal myxoid change and a mixed inflammatory infiltrate.

In 2004, Folpe and Weiss described 41 PHATs and recognized areas indistinguishable from HFLT at the periphery of many PHATs [4]. They coined the term “early PHAT” for these putative PHAT precursors [4]. The relationship between HFLT and PHAT was further strengthened by genetic studies highlighting either t(1;10)(p22;24) fusions or rearrangements of the *TGFBR3* and/or *MGEA5* genes in both tumor types [5–7]. The term “hybrid HFLT/PHAT” refers to tumors with features of both entities [8].

Several HFLT/PHATs (either primary or recurrent) showing morphologic progression to high-grade spindle cell sarcoma have been reported [9–13]. Most such cases have displayed features of myxofibrosarcoma [9, 10] or they vaguely resembled myxoinflammatory fibroblastic sarcoma [13].

Herein we report an exceptional example of hybrid HFLT/PHAT showing progression to a rapidly lethal, SMARCA4-deficient sarcoma with epithelioid and rhabdoid features, a previously undescribed phenomenon.

## Case history

**Fig. 1:**
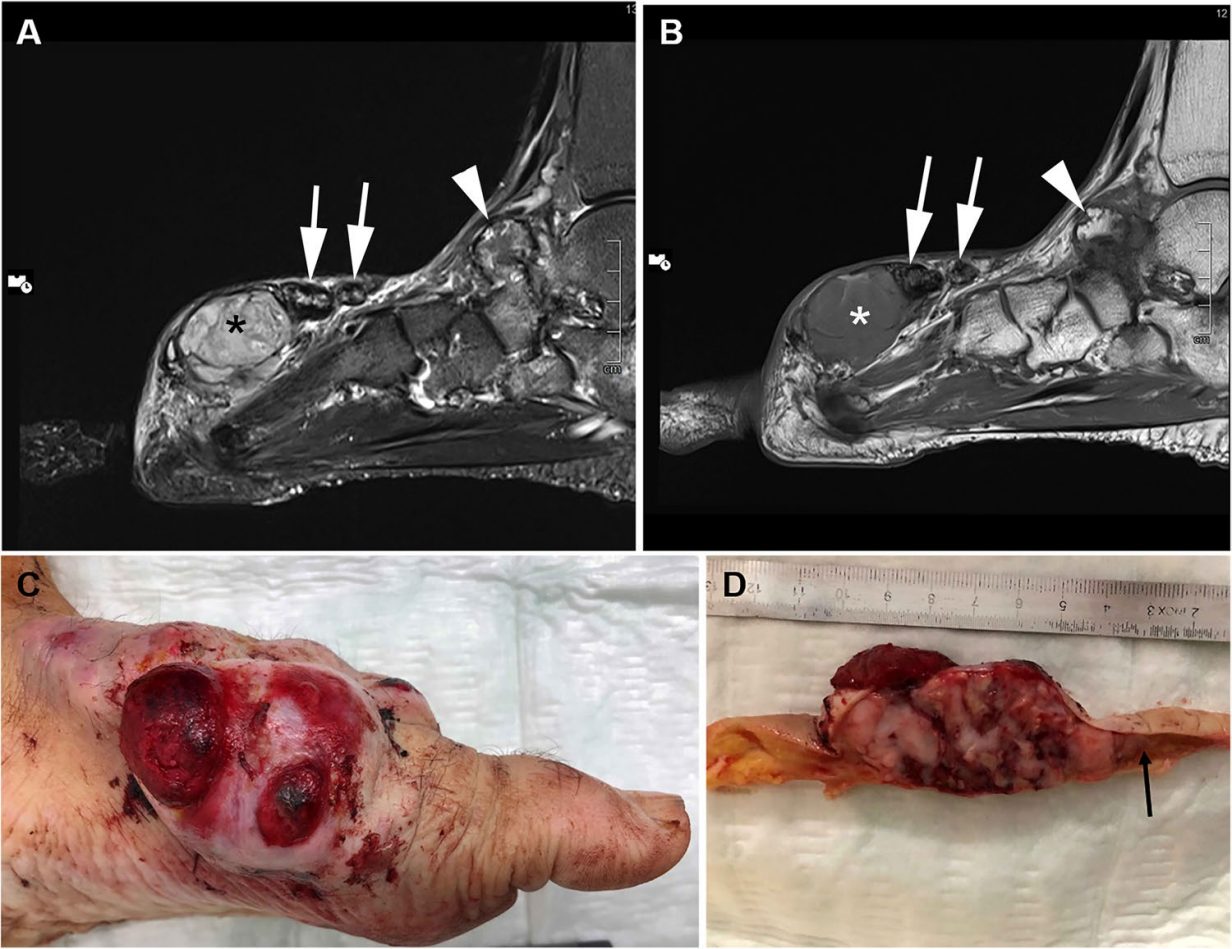
Representative examples of the imaging, clinical and gross findings. Sagittal fat suppressed (fs) T2-weighted MRI (**A**) shows a subcutaneous multi-lobulated mass of the midfoot dorsum. The lesion is well-demarcated by a hypointense rim and of heterogeneous high signal intensity (black asterisk). In addition there are smaller oval-shaped lesions continuous with the main mass exhibiting a hypointense rim in T1 and T2 fs reflecting hemosiderin deposits (arrows in **A** and **B**). The corresponding T1 weighted image shows heterogeneous hypointensity of main lesion (asterisk in **B**). A large osteophyte or osteoproliferation due to severe talonavicular osteoarthritis not related to the mass is highlighted by arrowheads in **A** and **B**. **C** Extensively ulcerated large multinodular mass bulging from the dorsum and medial aspect of the foot. **D** Cut-surface shows soft fleshy tan to reddish mass with extensive areas of necrosis and hemorrhage. Note brownish tissue on the right corresponding to the HFLT component (arrow)

A 54-year-old man with a 19-year history of rheumatoid arthritis, treated with methotrexate and etanercept, presented with a rapidly enlarging nodule on the dorsum of his foot. This mass had been present for 5 years and was clinically thought to represent a rheumatoid nodule. A nonradical surgical resection was performed at another center, and the patient was then referred to our sarcoma center for further treatment. Although the initial CT scan performed immediately after surgery showed minimal gross residual disease, the mass rapidly regrew, necessitating foot amputation (Fig. 1A, B). Post-operative staging CT revealed new, multiple, rapidly growing inguinal, pulmonary, and mediastinal/pleural metastases. Palliative chemotherapy was started with Adriamycin and ifosfamide, but his metastatic tumors continued to grow during the first and second cycles of chemotherapy. He then was put on immune checkpoint inhibition therapy with pembrolizumab, without response, and subsequently switched to trabectedin. However, his inguinal and mediastinal metastases continued to progress, and new lung metastases appeared. Due to rapidly progressive disease despite diverse therapeutic trials, treatment was terminated and the patient died 5 months after amputation.

## Material and methods

The tumor specimens were fixed in buffered formalin and embedded for routine histological examination. Immunohistochemistry (IHC) was performed on 3-µm sections using a fully automated system (“Benchmark XT System”, Ventana Medical Systems Inc, 1910 Innovation Park Drive, Tucson, AZ, USA) and the following antibodies: keratin cocktail (clones AE1/AE3, 1:40, Zytomed), desmin (clone D33, 1:250, Dako), alpha smooth muscle actin (clone 1A4, 1:200, Dako), gp100 protein (clone HMB45, 1:50, Enzo), ERG (EPR3864, prediluted, Ventana), CD30 (clone Ber-H2, 1:40, Zytomed), WT1-c-terminus (polyclonal, 1:50, Santa Cruz), S100 protein (polyclonal, 1:2500, Dako), synaptophysin (clone SY38, 1:50, Dako), CD34 (clone BI-3C5, 1:200, Zytomed), S100 protein (polyclonal, 1:2500, Dako), SOX10 (polyclonal, 1:25, DCS), MUC4 (EP256, 1:500, Epitomics), anti-NUT (clone C52B1, 1:45, Cell Signaling), SMARCB1 (INI1) (MRQ-27, 1:50, Zytomed), SMARCA2 (polyclonal antibody, 1:100, Atlas Antibodies AB), SMARCA4 (anti-BRG1 antibody, clone EPNCIR111A, 1:100, Abcam), ARID1A (rabbit polyclonal antibody, ab97995, 1:100; Abcam), and PBRM1 (clone CL0331; 1:50; Atlas Antibodies AB). Samples were used in accordance with ethical guidelines for the use of retrospective tissue samples provided by the local ethics committee of the Friedrich-Alexander University Erlangen-Nuremberg (ethics committee statements 24.01.2005 and 18.01.2012).

### Molecular testing

The tumor sample was tested for gene fusions using the TruSight RNA Panel (Illumina, Inc., San Diego, CA, USA) and for pathogenic mutations using the TruSight Tumor 170 Panel (Illumina) as described previously [14]. FISH testing for *TGFBR3* and *MGEA5* rearrangements was performed using previous published protocols [6].

### Pathological findings

Grossly, the amputation specimen showed extensive, multinodular mass on the dorsum of the foot with ulceration (Fig. 1C). On cut section, a highly fragile, necrotic, fleshy mass measuring 11 cm in maximum dimension was seen infiltrating through skin and subcutaneous tissue, and invading bone (Fig. 1D).

**Fig. 2:**
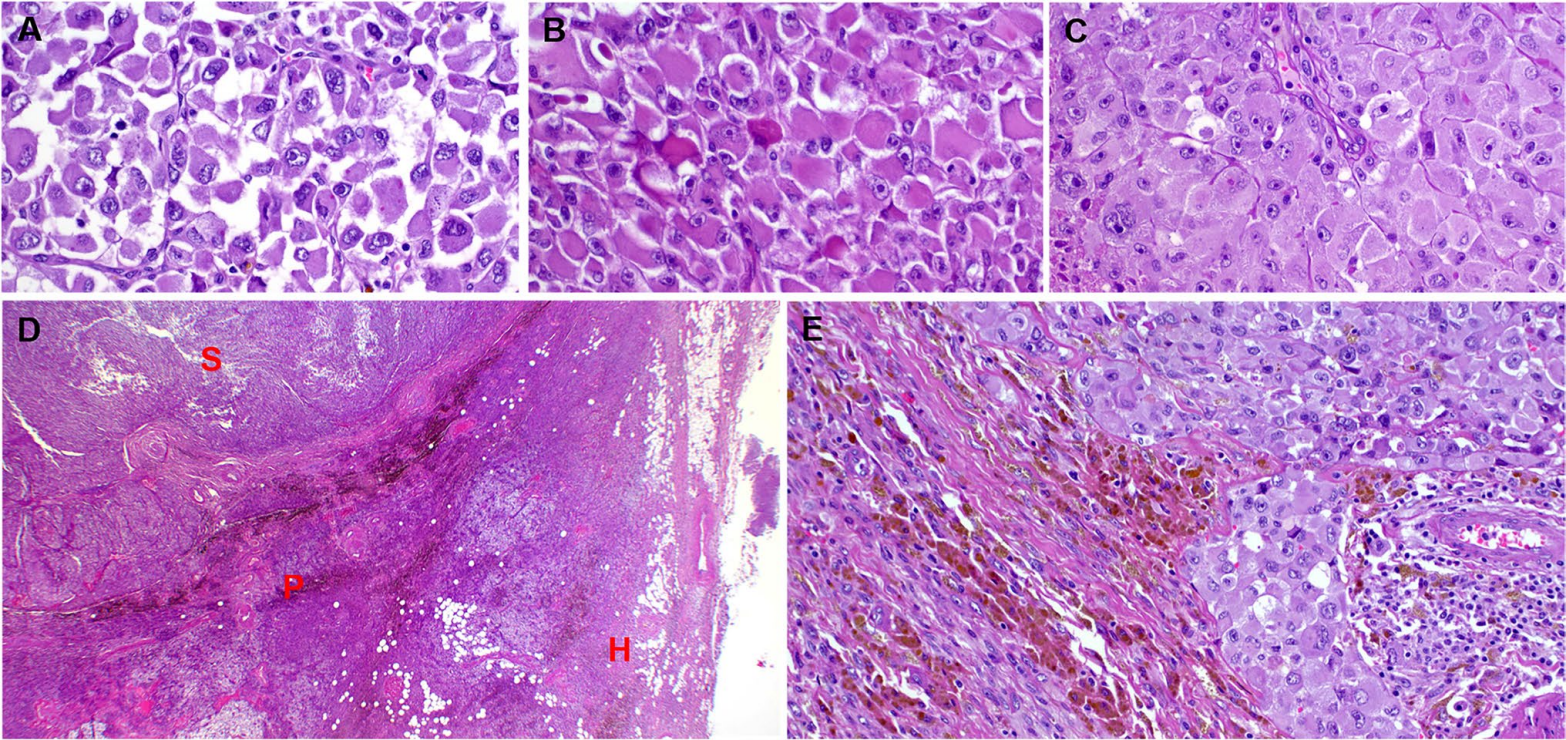
Histological findings of undifferentiated sarcoma ex HFLT/PHAT. At high power, the sarcoma cells range from poorly cohesive large epithelioid cells with ganglion cell-like features (**A**) to eosinophilic cells with copious rhabdoid cytoplasm (**B**) or densely packed large epithelioid cells (**C**). **D** The sarcoma component (S) is closely juxtaposed to the adjacent PHAT (P) component followed by prominent peripherally located HFLT component (H). **E** Focal blending of the undifferentiated sarcoma (right) and the HFLT component (left)

Histologically, the majority of the lesion (> 80%) was composed of an extensively necrotic, high-grade undifferentiated malignancy composed of poorly cohesive, large epithelioid and rhabdoid cells, growing in solid sheets and poorly cohesive aggregates (Fig. 2A–C). The tumor cells had vesicular nuclei with prominent centrally located macronucleoli, and were occasionally multinucleated with prominent rhabdoid features (Fig. 2B). Numerous mitotic figures, including atypical forms, were present.

**Fig. 3:**
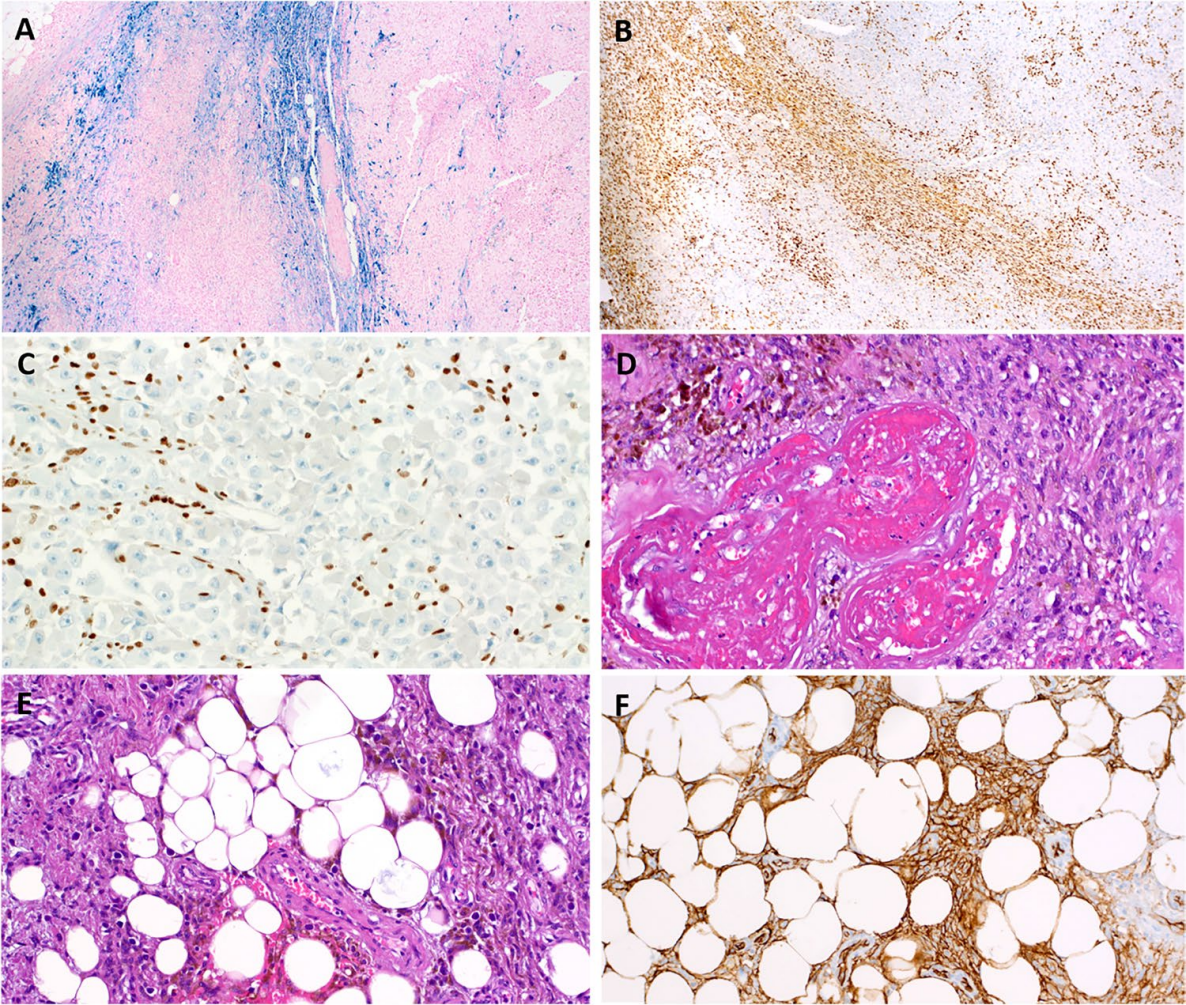
Detailed features of the HFLT/PHAT component. **A** The Prussian blue stain highlights the HFLT/PHAT component and sparing the sarcoma component (on right and focally on left). **B** Overview of the SMARCA4 immunohistochemistry; note the distribution of retained SMARCA4 expression in the HFLT/PHAT component, which is very similar to the Prussian blue pattern in **A**. **C** Higher magnification of SMARCA4 loss in the sarcoma component. **D** Higher magnification of the PHAT area. **E** Higher magnification of the HFLT at the periphery of the tumor. **F** CD34 in the HFLT area

The periphery of the mass was notable for a distinctly different-appearing second component, showing characteristic morphologic features of HFLT/PHAT, including abundant mature fat, fascicles of hemosiderin-laden spindled cells, ectatic, fibrin-filled blood vessels, and pleomorphic cells with nuclear pseudoinclusions (Fig. 2D, E). The undifferentiated high-grade tumor appeared to arise directly from the HFLT/PHAT-like areas, sometimes with “transitional” areas containing an increased number of atypical spindled cells (Fig. 2D, E and Fig. 3A, D–F). Reevaluation of the initial non-radical resection specimen revealed similar histology, containing both tumor components.

### Immunohistochemical findings

Because of the striking rhabdoid features of the high-grade component, the tumor was tested for expression of various components of the SWI/SNF complex (SMARCA4, SMARCA2, SMARCB1, ARID1A, and PBRM1); > 95% loss of SMARCA4 expression was present and was limited to the rhabdoid high-grade component, with retained expression of the other SWI/SNF subunits (Fig. 3B, C). Strong CD34 expression was observed in the HFLT and PHAT-like peripheral zones (Fig. 3F), but not in the high-grade sarcoma. Both components were negative with melanocytic, myogenic, and other lineage-specific markers.

### Molecular findings

The RNA fusion panel revealed no detectable fusions. Assessment of the expression results of the genes included in the panel showed highly overexpressed FGF8 with 809 reads compared to an expected median read of 0.40 counts in other tumor entities (data not shown). Elevated read counts were also detected for TGFBR3 (6682 reads compared to median count of 169), and MGEA5 (5158 reads compared to median count of 1111).

FISH analysis using *MGEA5* and *TGFBR3* probes confirmed rearrangements of both gene loci. Notably, both cell types (the spindled cells of the HFLT/PHAT component and the large epithelioid cells) showed split signals. The TST170 DNA Panel revealed no pathogenetic mutations in any other tumor-related genes. However, SMARCA4 is not included in this panel.

## Discussion

Sarcomas arising from pre-existing HFLT/PHAT are exceptionally rare, with fewer than 10 reported cases [9–13]. As noted above, such sarcomas typically show undifferentiated spindle cell morphology, with variable myxoid change and chronic inflammation.

The current case is morphologically quite distinct from previously reported cases, showing instead strikingly rhabdoid and epithelioid features, and displaying loss of SMARCA4 expression. Interestingly, this morphology is essentially identical to that seen in a variety of recently reported SMARCA4-deficient malignancies of diverse organs. Unlike SMARCB1 (INI1) loss, which is most often an “entity-defining” event in tumors such as epithelioid sarcoma and pediatric rhabdoid tumors, SMARCA4 loss occurs more often as a late genetic event driving dedifferentiation in carcinomas of various types [15], but also as a rare primary genetic event in histogenetically diverse entities [16, 17]. While the former often harbor several heterogeneous additional gene mutations [18], the latter usually display no gene mutations other than SMARCA4 inactivation [16, 17]. Lack of additional gene mutations in 170 commonly cancer-related genes in the current case argues for SMARCA4 loss being the major driver of transformation. In the present case, morphologic, immunohistochemical, and molecular- genetic testing clearly established a pre-existing HFLT/PHAT as the precursor lesion for this SMARCA4-deficient sarcoma. In the absence of areas of HFLT/PHAT, this lesion would be indistinguishable from rare variants of epithelioid sarcoma or a metastasis from a SMARCA4-deficient malignancy elsewhere. As with other SMARCA4-deficient sarcomas, the present tumor was highly aggressive, refractory to chemotherapy, and rapidly lethal.

As noted previously, HFLT and PHAT show many similar morphologic features and frequently demonstrate either t(1;10)(p22;q24) fusion or rearrangements of the *TGFBR3* (chromosome 1p22) and/or *MGEA5* (chromosome 10q24) loci [6]. Rearrangements involving *FBXW14* (10q24.2) have also been very recently reported [19]. Notably, the *TGFBR3-MGEA5* fusion seems to act via enhancer-related mechanisms resulting in altered gene expression without forming a detectable chimeric fusion protein. This may help to explain why the present tumor was FISH-positive, but negative by targeted RNA sequencing. In some cases, overexpression of FGF8 may serve as a “surrogate,” implying the presence of underlying *TGFBR3-MGEA5* fusion [19].

In summary, we have reported a case of SMARCA4-deficient undifferentiated sarcoma with rhabdoid/epithelioid features, arising from a pre-existing HFLT/PHAT of the foot. The distinctive morphology of this case, as compared to previously reported cases, likely reflects differences in the secondary genetic event (superimposed on pre-existing *TGFBR3/MGEA5* rearrangements). Although only limited conclusions can be drawn from a single case, the natural history of SMARCA4-deficient sarcoma arising in this unique setting appears to be identical to that of SMARCA4-deficient malignancies elsewhere, with significant potential for adverse patient outcome.
